# Genetic Diversity of Bacterial Communities and Gene Transfer Agents in Northern South China Sea

**DOI:** 10.1371/journal.pone.0111892

**Published:** 2014-11-03

**Authors:** Fu-Lin Sun, You-Shao Wang, Mei-Lin Wu, Zhao-Yu Jiang, Cui-Ci Sun, Hao Cheng

**Affiliations:** 1 State Key Laboratory of Tropical Oceanography, South China Sea Institute of Oceanology, Chinese Academy of Sciences, Guangzhou, China; 2 Daya Bay Marine Biology Research Station, South China Sea Institute of Oceanology, Chinese Academy of Sciences, Shenzhen, China; Universiteit Utrecht, Netherlands

## Abstract

Pyrosequencing of the 16S ribosomal RNA gene (rDNA) amplicons was performed to investigate the unique distribution of bacterial communities in northern South China Sea (nSCS) and evaluate community structure and spatial differences of bacterial diversity. Cyanobacteria, Proteobacteria, Actinobacteria, and Bacteroidetes constitute the majority of bacteria. The taxonomic description of bacterial communities revealed that more Chroococcales, SAR11 clade, Acidimicrobiales, Rhodobacterales, and Flavobacteriales are present in the nSCS waters than other bacterial groups. Rhodobacterales were less abundant in tropical water (nSCS) than in temperate and cold waters. Furthermore, the diversity of Rhodobacterales based on the gene transfer agent (GTA) major capsid gene (*g5*) was investigated. Four *g5* gene clone libraries were constructed from samples representing different regions and yielded diverse sequences. Fourteen *g5* clusters could be identified among 197 nSCS clones. These clusters were also related to known g5 sequences derived from genome-sequenced Rhodobacterales. The composition of *g5* sequences in surface water varied with the g5 sequences in the sampling sites; this result indicated that the Rhodobacterales population could be highly diverse in nSCS. Phylogenetic tree analysis result indicated distinguishable diversity patterns among tropical (nSCS), temperate, and cold waters, thereby supporting the niche adaptation of specific Rhodobacterales members in unique environments.

## Introduction

The bacterioplankton phylotypes of α-Proteobacteria are among the largest heterotrophic marine bacteria and often detected in various marine regions on Earth [1], [2]. Studies on marine microbial populations have suggested that Order Rhodobacterales (α-Proteobacteria) members are ubiquitous in marine environments and can account for >25% of total marine bacterioplankton [2]–[4]. Although Rhodobacterales has also been found as most abundant members in temperate and cold waters [5], [6], Rhodobacterales in tropical waters have been rarely investigated.

The complete genome sequences of Rhodobacterales contain gene transfer agent (GTA) gene clusters [7], [8]; these genes are not found in other major bacterioplankton groups. GTA is a small phage-like particle released by bacteria; each particle contains a random ca. 4.5 kb fragment of bacterial genomic DNA [9] that can be transferred between cells [10]. GTAs are present in phylogenetically diverse prokaryotes, indicating that this mode of DNA transfer may be important in shaping microbial genomes and communities [6]. GTA-related gene transfer has also been considered as a potential adaptive mechanism of these bacteria to maintain metabolic flexibility in changing marine environments [10], [11]. A capsid protein-encoding gene (*g5*) of GTA has been used as a marker to estimate the diversity of Rhodobacterales in temperate and cold waters because GTA genes are conserved in Rhodobacterales [5], [6].

Northern South China Sea (nSCS) is a marginal sea encompassing the Pearl River Estuary and a broad continental shelf. nSCS is characterized by tropical and subtropical climate and represents typical oligotrophic characteristics with significant environmental gradients from the discharge of the Pearl River; physical forces, such as mesoscale eddies, monsoon, upwelling, Kuroshio Current, and so on, influence nSCS [12]. All of these physical disturbances can influence water-column stability in different temporal and spatial scales [13]. Furthermore, nSCS consists of various ecosystems (such as mangrove forests, seagrass beds, coral reefs) marked with high biodiversities. However, the roles of heterotrophic bacterioplankton in these waters have not been explicitly characterized.

Although the distribution of the Rhodobacterales community in cold waters and temperate coast has been reported [2], [5], [6], [14], [15], the members of Rhodobacterales in tropical waters have not been described in detail. This study aimed to (i) determine the bacterial community and relative abundance of Rhodobacterales in nSCS, (ii) analyze the diversity and spatial genetic variations of the *g5* gene in nSCS, and (iii) compare *g5* structure of the nSCS with those from other areas.

## Methods

### Study stations and water sampling

**Figure 1:**
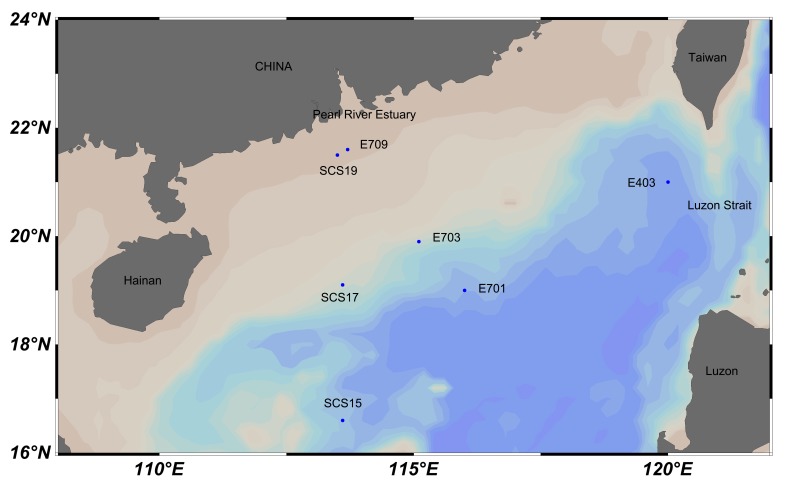
Map of sampling stations in the northern South China Sea.

E701, E703, E709, E403, SCS15, SCS17, and SCS19 are sampling stations in the South China Sea. Water samples (E701, E703, E709, and E403) were collected in September 2011. Samples of SCS15, SCS17, and SCS19 were collected in May 2013 (Figure 1). Water samples at each station were collected and 1000 mL of seawater was filtered with 0.22 µm pore size filters (47 mm in diameter, Millipore Corp., Bedford, USA) at low vacuum pressure to collect prokaryotic cells. Each sample was prepared in three replicates. After filtration was performed, the membranes were immediately frozen in liquid nitrogen and then stored at −20°C until DNA extraction was conducted in our laboratory.

### Ethics statement

No specific permits were required for the described field studies. Our study area is not privately owned or protected in any way. Our field studies did not involve endangered or protected species. The South China Sea Institute of Oceanology and Chinese Academy of Sciences issued the permissions to investigate each location.

### DNA extraction, PCR amplification, and pyrosequencing

For each sample, triplicate DNA aliquots were extracted according to the special DNA protocol for marine bacterial communities [16]. A region of 444 bp in the 16S rRNA gene covering the V1–V3 region was selected to construct a community library by tag pyrosequencing. The broadly conserved bar-coded primers 27F and 533R containing A and B sequencing adaptors (454 Life Sciences) were used to amplify this region. The forward primer (B-27F) sequence was 5′-*CCTATCCCCTGTGTGCCTTGGCAGTCTCAG*AGAGTTTGATCCTGGCTCAG-3′, in which the B adaptor sequence is italicized and underlined. The reverse primer (A-533R) sequence was 5′-*CCATCTCATCCCTGCGTGTCTCCGACTCAGNNNNNNNN*TTACCGCGGCTGCTGGCAC-3′, in which the sequence of the A adaptor is italicized and underlined. Ns represent an eight-base sample-specific barcode sequence. Amplicon pyrosequencing was performed from the A-end by using a 454/Roche A sequencing primer kit on a Roche Genome Sequencer GS FLX Titanium platform at Majorbio Bio Tech Co. Ltd (Shanghai, China). We eliminated sequences that contained more than one ambiguous nucleotide and a primer at one end or sequences that were shorter than 200 bp after barcode and primer sequences were removed. Pyrosequencing reads were simplified using the ‘unique.seqs’ command to generate a unique set of sequences. These pyrosequencing sequences were aligned using the ‘align.seqs’ command and compared with the Bacterial SILVA database (SILVA version115, http://www.arb-silva.de). Aligned sequences were trimmed further and redundant reads were eliminated using the ‘screen.seqs’, ‘filter.seqs’, and ‘unique.seqs’ commands in that order. The ‘chimera.slayer’ command was used to determine chimeric sequences. The ‘dist.seqs’ command was used, and unique sequences were assigned to operational taxonomic units (OTUs, 97% similarity). In the present study, data preprocessing and OTU-based analysis were performed on Mothur [17]. Taxonomic assignments with <80% confidence were marked as unknown. All of the sequences can be downloaded from the NCBI Sequence Read Archive database under the accession numbers SRX547142-SRX547144.

### PCR amplification and clone library analyses of GTA *g5* genes

The primers used to amplify GTA *g*5 genes described in a previous study [5] were used in the present study. These primers include MCP-109F, 5′-GGC TAY CTG GTS GAT CCS CAR AC-3′ and MCP-368R, and 5′-TAG AAC AGS ACR TGS GGY TTK GC-3′. Target DNA was amplified in a single round of PCR in reaction volumes of 50 µl containing 10 pmol of each primer, 4 µL of 5 mM dNTPs, 1.25 U of *Taq* DNA polymerase (Takara, Japan), and 3% DMSO (v/v). The thermocycling conditions used in this study were listed as follows: 5 min at 95°C; 35 cycles at 95°C for 30 s, 60°C for 30 s, and 72°C for 30 s; and a final extension step at 72°C for 7 min.

The purified PCR products of *g*5 genes were inserted into the pMD-18T vector (Takara, Japan) to construct clone libraries. Positive clones were selected to sequence and analyze using an ABI3730 DNA sequencer. All of the *g5* sequences were edited using CROSS-MATCH to remove vector and primer sequences [18]. The DNA sequences were subsequently translated into an amino acid sequence. The resulting capsid protein sequences obtained in this study were aligned and compared with the reference sequences in the GenBank database. Neighbor-joining phylogenetic trees were constructed using the MEGA 5.0 software [19]. Evolution distances were calculated using Jones-Taylor-Thornton model with a rate variation among sites and complete gap deletions to translate the *g5* gene sequence into its corresponding amino acid sequence [5]. The sequences obtained from the four clone libraries were deposited in the GenBank database with the accession numbers of KC422732 to KC422774.

The aligned sequences in each clone library were analyzed using Mothur software [17] to determine operational taxonomic units (OTUs) at a 3% dissimilarity cut-off. Simultaneously, Mothur was used to estimate the richness indices (Chao1 and Shannon), diversity index (Simpson), and coverage [17]. The structure of g5 genes was analyzed using Euclidean distance by multi-dimensional scaling (MDS) analysis in SPSS 18.0 for Windows. MDS is an ordination technique that represents the samples as points in a multi-dimensional space. Sample communities with the highest similarity in the data set are shown as the closest plotted points, and the communities with the lowest similarity are indicated by the points that are the farthest apart.

To directly assess the relationship between the structure of the *g5* gene and water environment of the nSCS, a canonical correspondence analysis (CCA) was carried out using the CANOCO 4.5 for Windows [20]. Statistical significance (at the 5% level) of relationships between *g5* gene data and environmental variables were assessed using the Monte Carlo permutation test (499 permutations).

## Results

### Taxonomic composition analysis

A total of 31,831 valid sequences and 3,392 OTUs (1331, 1321, and 1340) were obtained from the three samples (SCS19, SCS17, and SCS15) by 454 pyrosequencing analyses; among these sequences, two reads corresponded to eukaryotes and were excluded in the subsequent analyses. The remaining sequences were then assigned to 15 different phyla or groups.

**Figure 2:**
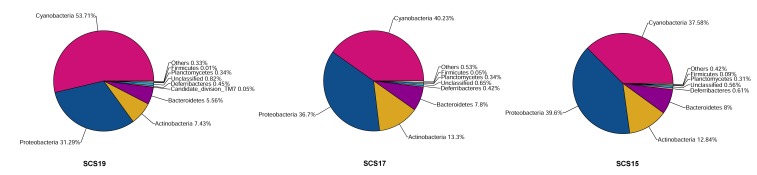
Bacterial compositions of the different samples in the nSCS.

The three samples showed similar bacterial community distributions in phylum level (Figure 2). Overall, the most abundant groups in surface water were affiliated to the phylum Cyanobacteria, which represented 43.58% of the pyrosequencing tags. The second most abundant group was Proteobacteria (35.97%), which were mainly Alphaproteobacteria (32.33%), followed with Actinobacteria (11.29%) and Bacteroidetes (7.16%).

**Figure 3:**
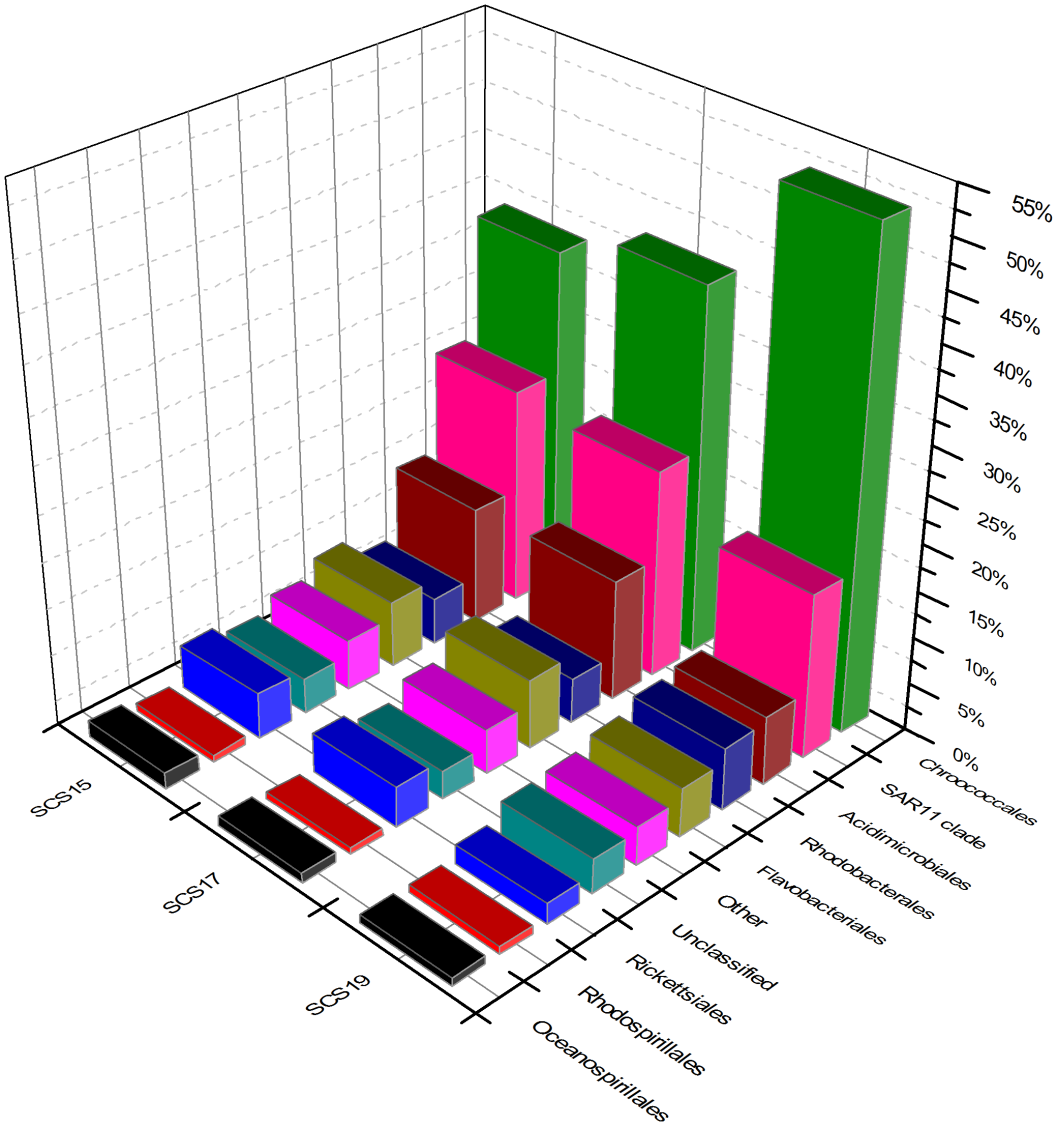
Bacterial compositions of the different communities in the nSCS.

From the area near the shore station to the far sea area, the Cyanobacteria group decreased sharply from 53.71% in SCS19 to 37.58% in SCS15. By contrast, Proteobacteria, Actinobacteria, and Bacteroidetes content increased gradually from SCS19 to SCS15 (Figure 2). In the order level (Figure 3), the Chroococcales group had the highest percentage of specific taxonomy in SCS19, SCS17, and SCS15 with 51.75%, 39.23%, and 35.94%, respectively. The Chroococcales group in SCS19, SCS17, and SCS15 consisted mainly of *Synechococcus* (30.67%, 5.90%, and 9.23%) and *Prochlorococcus* (20.22%, 32.76%, and 26.01%). Other bacterial orders that dominated the samples included SAR11 clade (17.48%, 22.23%, and 23.17%), Acidimicrobiales (7.14%, 12.83%, and 12.46%), Flavobacteriales (5.28%, 7.38% and 7.10%), and Rhodobacterales (6.67%, 4.75%, and 4.89%). Other groups were rare and many sequences were present at abundance <1% of the total population.

Among all of the Rhodobacterales sequences, only 9% sequences could be determined to identify genus. In the bacterial genus level, bacterial groups related to Rhodobacterales from the nSCS waters were identified by phylogenetic tree analysis. These groups were diverse: *Donghicola*, *Labrenzia*, *Loktanella*, *Maritimibacter*, *Paracoccus*, *Pelagibaca*, *Rhodovulum*, *Roseobacter*, *Ruegeria*, *Thalassococcus*, and *Citreicella* (Figure S1).

### Diverse and unique *Rhodobacterales* in the nSCS

**Figure 4:**
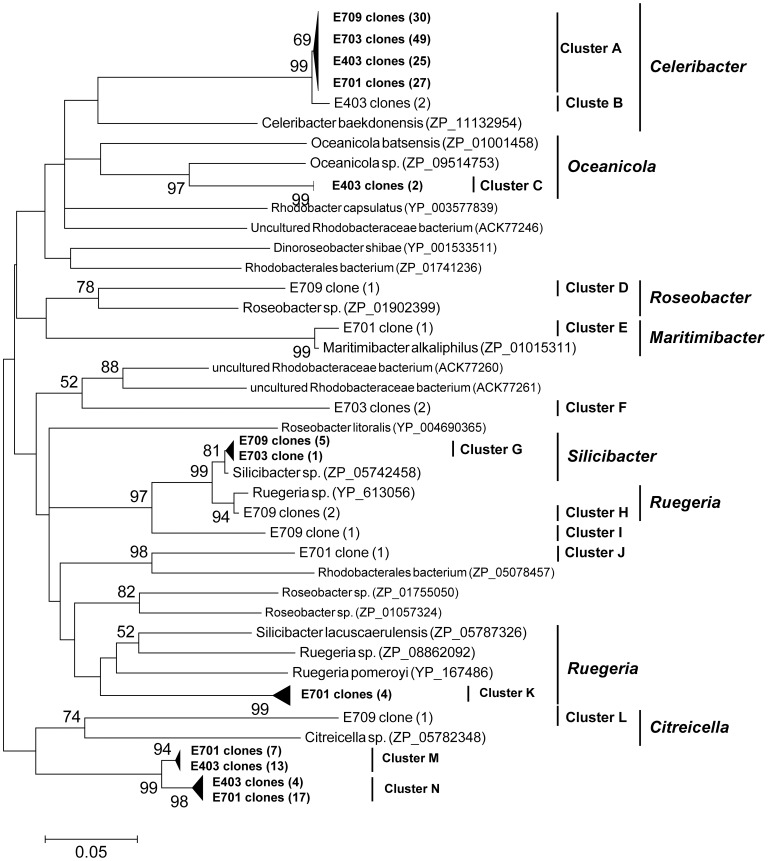
Neighbor-joining phylogenetic tree based on partial *g5* amino acid sequences (ca. 250 aa) showing the phylogenetic diversity of *g5* in the northern South China Sea.

GTA diversity was assessed in the four samples representing different regions (E709, E703, E701, and E403). A total of 197 sequences were recovered from these four clone libraries. The phylogenetic analysis of the g5 clone sequences fell within the Rhodobacterales and corresponded to 14 phylogenetic clusters (designated as A–N; Figure 4).

The coverage of four clone libraries ranged from 85.7% to 98.1% at the 3% distance cut-off, indicating that clone libraries adequately covered the diversity of *g5* genes (Table S1). Shannon–Weaver and Simpson indices revealed that *g5* gene diversity was higher in site E701 than in sites E709, E703, and E403. Chao 1 demonstrated that the richness at sample E701 was greater than that at other samples. However, low Simpson index was observed in all samples (Table S1).

**Figure 5:**
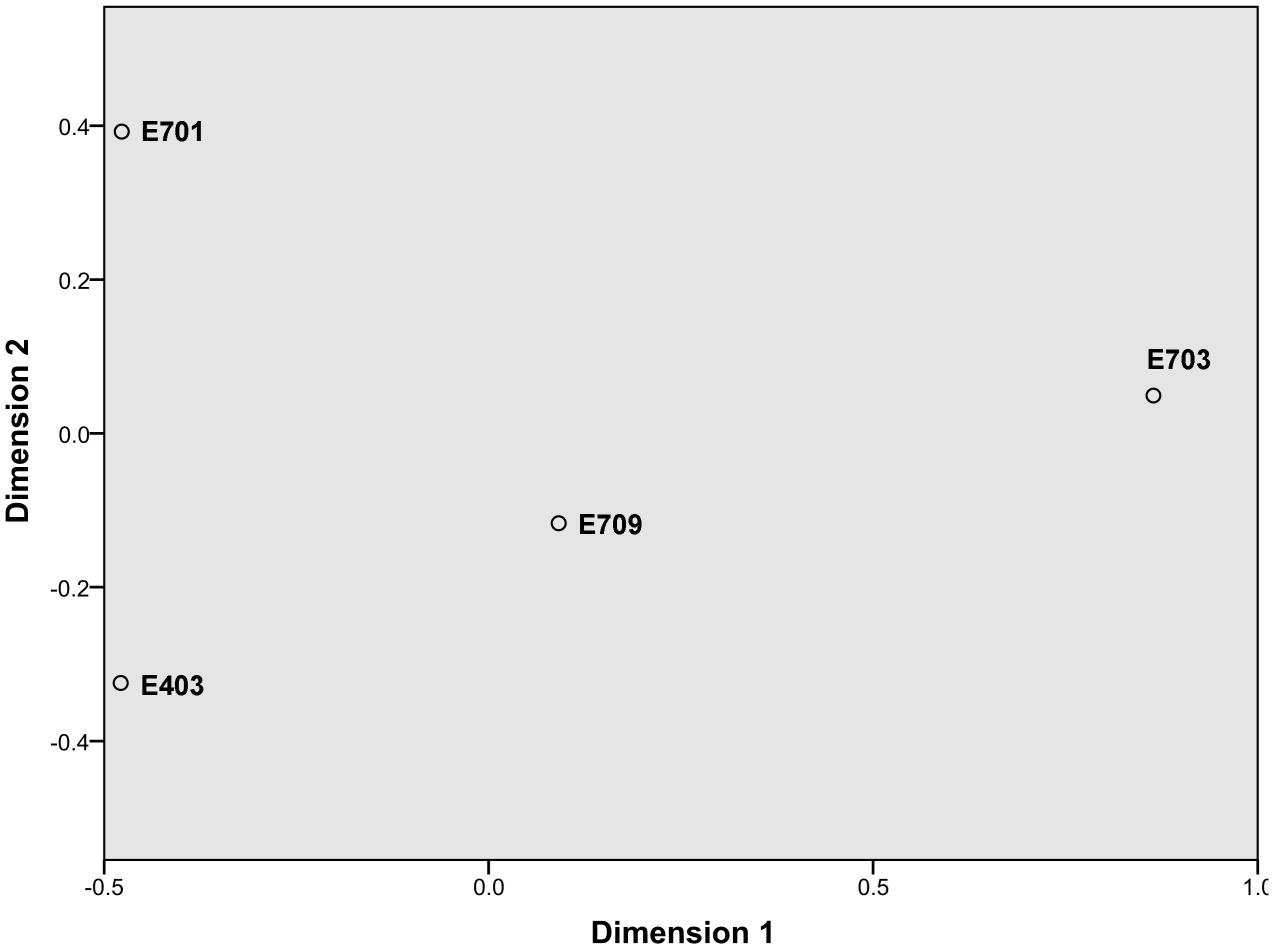
Two-dimensional plots of MDS analysis from *g5* gene clone library to compare broad-scale differences between Rhodobacterales communities.

Two-dimensional plots of MDS for samples showed a spatial diversity in the *g*5 gene structure (Figure 5). The results revealed that four plots that represented *g*5 structure from samples E701, E703, E709, and E403 had large distances with one another and had an MDS stress value of 0.02. Stress values below 0.2 indicate that an MDS ordination plot is a good spatial representation of differences between data. Overall, MDS ordination plots indicated that the composition of the *g*5 structure varied with the sampling sites (ANOVA, p<0.01).

The CCA of the *g5* gene data explained 75.3% of the variation in the first two axes (Figure S2). According to Monte Carlo analysis, only latitude (F = 1.62, P = 0.038) showed a significant correlation to the *g5* gene structure. By contrast, other environmental factors (temperature, salinity, and Chla) had no significant correlation to *g5* gene structure (p>0.05).

### Variation of GTA capsid genotypes in the nSCS

A spatial variation of *g*5 composition in the nSCS was evident (Figure 4, Table S1, Table S2). *g5* sequence data was grouped into 14 clusters, labeled A–N. Clusters D, H, I, and L were unique to E709 and closely related to *Roseobacter*, *Ruegeria*, and *Citreicella*. Cluster F was unique to E703 and closely related to uncultured Rhodobacteraceae bacterium. Clusters E, J, and K only appeared in site E709, and clusters E and K were closely related to *Maritimibacter* and *Ruegeria*. Clusters B and C only appeared in site E403 and were related to *Celeribacter and Oceanicola*. Cluster A constitutes more than 47% of the *g5* clones in four clone libraries, especially in E709 and E703, which was related to *Celeribacter*, achieved 76.19% and 94.23%. Cluster G was present in sites E709 and E703 and related to *Silicibacter*. Clusters M and N were present in sites E701 and E403; however, we did not find high matching sequences in the GenBank database.

Clusters D, H, I, and L accounted for 11.9% of the E709 clone library and were not detected in other libraries (Figure 4, Table S3). Station E703 had the lowest *g5* diversity among the investigation stations. The highest number of clones in cluster A was observed in station E703 among all of the other libraries; this result suggested that *Celeribacter* was dominant in Rhodobacterales bacteria (Figure 4). Clusters B and C in site E403 were more representative of a typical Rhodobacterales cluster and may have been derived from the external area of the Pacific Ocean. Station E701 had three common clusters (cluster A, M, and N) with E403 and only one common cluster (cluster A) with E709.

### Comparison of *g5* structure of the nSCS with those from other areas

**Figure 6:**
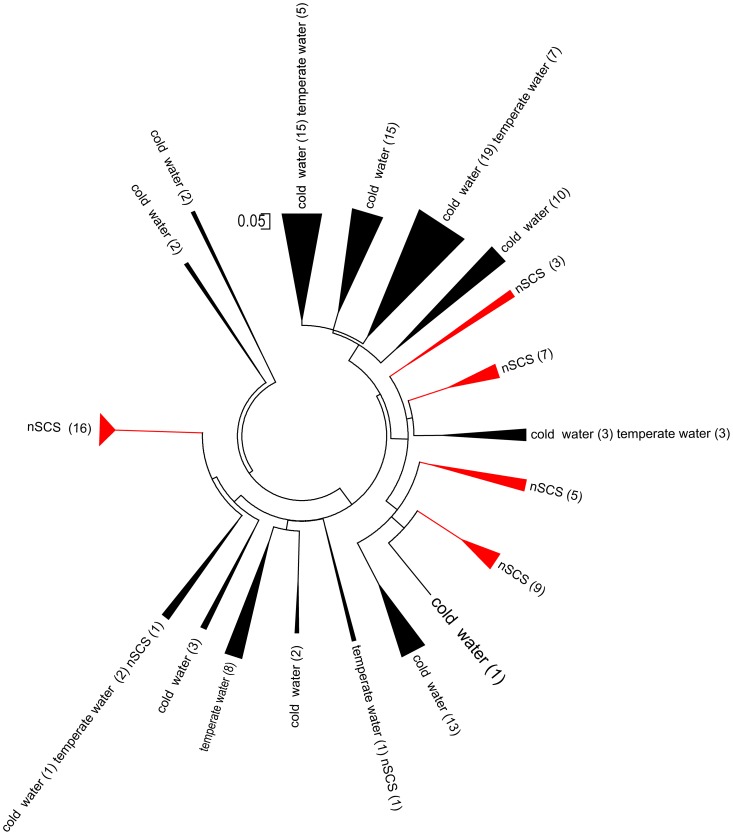
Biogeographic distribution of *g5* gene OTUs in the nSCS (red) compared with those from Subartic North Atlantic Ocean (cold water), the Arctic Ocean (cold water) and Chesapeake Bay (temperate water).

The *g*5 gene sequences that belonged to the uncultured environmental samples from the Subartic North Atlantic Ocean (cold water), the Arctic Ocean (cold water), and Chesapeake Bay (temperate water) were retrieved from the GenBank database. These sequences were aligned and analyzed with Mothur to determine the OTUs at a 3% dissimilarity cut-off. The resulting capsid protein sequences obtained in this study were aligned and compared with these 134 OTUs. Homology analysis was conducted to align the nSCS gene sequence (36 OTUs) with these *g*5 gene OTU sequences. A phylogenetic tree was constructed using MEGA5.0 for the translated amino acid sequence of the *g*5 gene (Figure 6). Our results showed that the majority of g5 sequences from temperate water were most similar to sequences obtained from Subartic North Atlantic and Atlantic Ocean waters. The *g5* genes in the nSCS had unique sequences, and the majority of the *g5* gene OTUs had no similarity to the *g5* gene OTUs from other regions. Furthermore, a few OTUs were similar to OTUs in temperate and cold ocean waters.

## Discussion

In many studies, Rhodobacterales abundance could reach above 25% through sequence analysis from the Atlantic Ocean to the Pacific Ocean [21]–[24]. These findings indicated that Rhodobacterales is the primary bacteria group in the cold and temperate water marine ecosystems. Thus far, studies focusing exclusively on Rhodobacterales in tropical water have not been reported.

In the current study, Rhodobacterales and other bacterial communities in the nSCS were assessed for the first time. Our results showed that the average abundance of Rhodobacterales was 5.44% for all the sequences. Although Rhodobacterales had a relatively higher abundance than other groups in nSCS waters, the Rhodobacterales content was lower than that from temperate and cold waters. Nevertheless, Rhodobacterales was one of the dominant orders of bacterial communities in nSCS.

Pyrosequencing analysis indicated that Cyanobacteria and Proteobacteria dominated the nSCS. The overwhelming majority of the identified Cyanobacteria sequences were related to *Synechococcus* and *Prochlorococcus*. These bacteria dominated the cyanobacterial communities in coastal and offshore station of the nSCS and were considered dominant groups in tropical ocean ecosystems. *Prochlorococcus* and *Synechococcus*, the most abundant photosynthetic microorganisms in oceans, contribute significantly to primary production [25], [26]. *Prochlorococcus* and *Synechococcus* are likely to contribute significantly to the primary production in the SCS because most of the nSCS exhibits oligotrophic characteristics.

Although *Synechococcus* and *Prochlorococcus* often occur simultaneously, they have different adaptation types depending on biogeochemical conditions. *Synechococcus* has also been reported to be abundant in environments with low salinities and/or low temperatures. *Synechococcus* is more abundant in nutrient-rich areas than in oligotrophic areas. Our results indicated that the abundance of *Synechococcus* decreased from 30.67% (SCS19) to 5.90% (SCS15). In the nSCS, a lower temperature (24.42°C) was detected in SCS19 than in SCS17 (28.97°C) and SCS15 (29.89°C). Temperature may have regulated the abundances of *Synechococcus* in nSCS waters. In contrast to *Synechococcus*, *Prochlorococcus* is generally absent in brackish or well-mixed waters and more abundant in warm oligotrophic areas, which correspond to a major part of the oceans on Earth [27]. The northern part of SCS has typical oligotrophic characteristics with significant environmental gradients from the discharge of the Pearl River. *Prochlorococcus* could adapt to oligotrophic environments and was more abundant in SCS17 (32.76%) and SCS15 (26.01%) than in SCS19 (20.22%).

SAR11 clade accounted for 17.48%–23.17% of rRNA genes that have been identified in the nSCS by pyrosequencing methods in our study. Bacteria belonging to the SAR11 clade frequently constituted 25% or more of the cloned 16S rRNA gene sequences retrieved from seawater samples around the world [28]. SAR11 bacteria were responsible for about 50% of the amino acid assimilation and 30% of the DMSP assimilation in surface waters because these bacteria are highly abundant and active [29]. The high abundance of SAR11 suggested that members of this clade could play an important role in C, N, and S cycling in nSCS. Significant correlations were observed between the abundance of SAR11 and the abundance of *Prochlorococcus* [30]. Both *Prochlorococcus* and SAR11 have maximized their ability to consume nutrients efficiently at very low nutrient concentrations [30], [31].

The pyrosequencing analysis results showed that the Rhodobacterales bacteria exhibited highly diverse sequences despite a relatively low abundance. In the bacterial genus level, Rhodobacterales from nSCS waters included 11 identified genera, which displayed high diversity. In the *g5* gene cluster, only six genera of Rhodobacterales were found. Most of these genera were included in the results of the 16S rRNA phylogenetic analysis. *g5* was highly conserved among all of the Rhodobacterales bacteria, and the phylogeny based on *g5* was consistent with that based on 16S rRNA genes [32]. Inconsistency phylogeny between *g5* and 16S rRNA gene was observed in this study possibly because of sampling site differences. This study also indicated that Rhodobacterales bacteria in the nSCS showed an evident spatial heterogeneity because of complex hydrographic conditions in nSCS (Table S2; Table S3). Most of the Rhodobacterales genera obtained in this study, such as *Roseobacter*, *Silicibacter*, and *Ruegeria*, could undergo aerobic anoxygenic photosynthesis, sulfur oxidation, carbon monoxide oxidation, and DMSP demethylation [3], [33]–[35]. These traits are important in the nSCS ecosystem because most parts belong to oligotrophic waters. The roles of Rhodobacterales in tropical waters should be investigated in future studies.

Most *g*5 clones in nSCS had low amino acid identities (<90%) compared with known g5 sequences that were derived from genome-sequenced Rhodobacterales. Although Rhodobacterales bacteria had high levels of 16S rRNA sequence similarities with known GenBank sequences, a lower similarity match was found when comparing *g5* gene sequences with GenBank database. This result also suggested that different Rhodobacterales bacteria contain highly diverse GTA genes in unique environments.

The diversity of the *g5* gene of Rhodobacterales was higher in the offshore stations (E403 and E701) than near shore stations (E703 and E709). A significant correlation between geographic distance (latitude) and *g5* compositions (p<0.05) was found when the relationship between location, temperature, salinity, and *g5* compositions was analyzed; by contrast, other environmental factors (temperature, salinity, and Chla) had no apparent effect on *g5* structure (p>0.05). Zhao et al. [5] also found that the composition of g5 sequences varies remarkably in different locations along the Chesapeake Bay. Furthermore, distinguishable diversity patterns are found between temperate and subarctic waters [6]. Geographic distance could be accounted for *g5* gene diversity differences in nSCS.

The g5 gene was observed to have different clusters among the sampling sites in the nSCS. Clusters D, H, I, and L of the E709 clone library were not detected in other libraries, suggesting that a unique Rhodobacterales may be present near shore water (Figure 4). Station E709 is located in the Pearl River Estuary, and inshore area input affects this station in wet season. The Rhodobacterales community of this station had a distinct structure compared with other sites. Site E403 is near the Luzon Straits, and the Pacific Ocean largely influences this station [36]. Clusters B and C may represent typical Rhodobacterales cluster better than the other clusters recovered in this study. This cluster may have been derived from the external Pacific Ocean water. Offshore stations represented E701, which is influenced by near shore water and Pacific Ocean water in geographically. The site E701 sample had one common *g5* gene cluster with site E709, and had three common *g*5 clusters with E403, which indicated that the Pacific Ocean water affected the Rhodobacterales composition in E701 to a great extent.

Studies on *g5* genetic diversity have mainly focused on cold and temperate waters, such as the Subartic North Atlantic Ocean [6], Arctic marine [15], and Chesapeake Bay [5]. The overwhelming majority of *g5* gene OTUs in the South China Sea was different from these areas. Only a few *g5* gene OTUs sequences in the South China Sea had a close genetic distance to the sequences from the cold and temperate waters. The results also indicated that the *g5* gene in the nSCS had a characteristic regional distribution. Furthermore, Shannon index in the nSCS (*H′* = 2.35) was similar to temperature water (*H′* = 2.39) [5] and lower than cold water (*H′* = 3.71) [6]. Overall diversity of the Rhodobacterales community, as inferred from g5 gene sequences, in the subarctic and arctic water appears higher than that in the temperate and tropical waters.

## Conclusions

The present study demonstrated the spatial distribution of bacterial communities in nSCS environments. The South China Sea had more Chroococcales, SAR11 clade, Acidimicrobiales, Flavobacteriales, and Rhodobacterales than other bacterial groups. Rhodobacterales exhibited high diversity in nSCS despite relatively low abundance. Differences of *g5* gene composition from tropical, temperate, and cold regions also suggested the specific adaptations of Rhodobacterales to different environments. Further research on the isolation and characterization of indigenous Rhodobacterales in nSCS may improve our understanding of the ecological roles of Rhodobacterales in tropical waters.

## Supporting Information

Figure S1 **Phylogenetic tree analysis based on partial 16S rRNA gene of Rhodobacterales in the northern South China Sea.**
(TIF)

Figure S2 **Canonical correspondence analysis (CCA) ordination diagram of** ***g5*** **gene composition in northern South China Sea, with environmental factors as arrow.**
(TIF)

Table S1 **Characteristics of environmental parameters and clone information for each sampling station.**
(DOC)

Table S2 **Comparison of** ***g*** **5 gene OTUs composition and distribution in four clone libraries.**
(DOC)

Table S3 **Comparison of g5 gene cluster and distribution in the nSCS.**
(DOC)
