# Supplementary material for: Genetic Diversity of Bacterial Communities and Gene Transfer Agents in Northern South China Sea
Source: PLoS One. 2014 Nov 3;9(11):e111892. doi: 10.1371/journal.pone.0111892 (PMC4218858; doi:10.1371/journal.pone.0111892)
Supplement: Table S1 — Characteristics of environmental parameters and clone information for each sampling station. (DOC) [file pone.0111892.s003.doc]

Table S1 Characteristics of environmental parameters and clone information for each sampling station

| Sites | Temperature (°C) | Salinity | Chl *a* (μg/L) | cutoff | Clone numbers | OTUs | Shannon | Simpson | Chao | Coverage (%) |
| --- | --- | --- | --- | --- | --- | --- | --- | --- | --- | --- |
| E709 | 28.96 | 33.68 | 0.7195 | 0.03 | 42 | 11 | 1.70 | 0.276 | 18.50 | 85.7 |
| E703 | 30.24 | 33.47 | 0.0948 | 0.03 | 52 | 5 | 1.04 | 0.422 | 5.00 | 98.1 |
| E701 | 30.19 | 33.67 | 0.0951 | 0.03 | 57 | 15 | 2.36 | 0.098 | 36.0 | 87.7 |
| E403 | 28.36 | 34.27 | 0.0782 | 0.03 | 46 | 12 | 1.93 | 0.195 | 13.0 | 91.3 |
