# Supplementary material for: Genetic Diversity of Bacterial Communities and Gene Transfer Agents in Northern South China Sea
Source: PLoS One. 2014 Nov 3;9(11):e111892. doi: 10.1371/journal.pone.0111892 (PMC4218858; doi:10.1371/journal.pone.0111892)
Supplement: Table S2 — Comparison of g 5 gene OTUs composition and distribution in four clone libraries. (DOC) [file pone.0111892.s004.doc]

Table S2. Comparison of *g*5 gene OTU composition and distribution in four clone libraries

|  | E709 | E703 | E701 | E403 |
| --- | --- | --- | --- | --- |
| OTU1 | 2 | 0 | 0 | 0 |
| OTU2 | 1 | 1 | 0 | 0 |
| OTU3 | 1 | 0 | 0 | 0 |
| OTU4 | 0 | 0 | 1 | 0 |
| OTU5 | 0 | 0 | 1 | 0 |
| OTU6 | 0 | 0 | 1 | 0 |
| OTU7 | 0 | 0 | 10 | 1 |
| OTU8 | 0 | 0 | 3 | 11 |
| OTU9 | 0 | 0 | 0 | 1 |
| OTU10 | 12 | 25 | 5 | 8 |
| OTU11 | 0 | 0 | 2 | 0 |
| OTU12 | 8 | 4 | 0 | 0 |
| OTU13 | 2 | 0 | 3 | 8 |
| OTU14 | 0 | 0 | 3 | 2 |
| OTU15 | 0 | 0 | 2 | 0 |
| OTU16 | 0 | 0 | 1 | 0 |
| OTU17 | 3 | 16 | 1 | 1 |
| OTU18 | 0 | 0 | 3 | 0 |
| OTU19 | 1 | 3 | 0 | 0 |
| OTU20 | 3 | 0 | 3 | 4 |
| OTU21 | 0 | 0 | 2 | 1 |
| OTU22 | 3 | 0 | 2 | 2 |
| OTU23 | 0 | 1 | 0 | 0 |
| OTU24 | 3 | 0 | 0 | 0 |
| OTU25 | 1 | 0 | 0 | 0 |
| OTU26 | 1 | 0 | 0 | 0 |
| OTU27 | 0 | 0 | 7 | 2 |
| OTU28 | 0 | 0 | 0 | 1 |
| OTU29 | 0 | 0 | 4 | 2 |
| OTU30 | 0 | 0 | 0 | 1 |
| OTU31 | 1 | 0 | 0 | 0 |
| OTU32 | 0 | 2 | 0 | 0 |
| OTU33 | 0 | 0 | 1 | 0 |
| OTU34 | 0 | 0 | 1 | 0 |
| OTU35 | 0 | 0 | 0 | 1 |
| OTU36 | 0 | 0 | 1 | 0 |
