# Supplementary material for: Genetic Diversity of Bacterial Communities and Gene Transfer Agents in Northern South China Sea
Source: PLoS One. 2014 Nov 3;9(11):e111892. doi: 10.1371/journal.pone.0111892 (PMC4218858; doi:10.1371/journal.pone.0111892)
Supplement: Table S3 — Comparison of g5 gene cluster and distribution in the nSCS. (DOC) [file pone.0111892.s005.doc]

Table S3 Comparison of g5 gene cluster and distribution in the nSCS

| Cluster | E709 | E703 | E701 | E403 |
| --- | --- | --- | --- | --- |
| A | 32 (76.19%)^a^ | 49 (94.23%) | 27(47.37%) | 25(54.35%) |
| B | 0 | 0 | 0 | 2(4.35%) |
| C | 0 | 0 | 0 | 2(4.35%) |
| D | 1(2.38%) | 0 | 0 | 0 |
| E | 0 | 0 | 1(1.75%) | 0 |
| F | 0 | 2(3.85%) | 0 | 0 |
| G | 5(11.90%) | 1(1.92%) | 0 | 0 |
| H | 2(4.76%) | 0 | 0 | 0 |
| I | 1(2.38%) | 0 | 0 | 0 |
| J | 0 | 0 | 1(1.75%) | 0 |
| K | 0 | 0 | 4(7.02%) | 0 |
| L | 1(2.38%) | 0 | 0 | 0 |
| M | 0 | 0 | 7(12.28%) | 13(28.26%) |
| N | 0 | 0 | 17(29.82%) | 4(8.70%) |

^a^The number of clones (and relative percentage) for a given cluster in the library.
